# The Information Length Concept Applied to Plasma Turbulence

**DOI:** 10.3390/e26060494

**Published:** 2024-06-05

**Authors:** Johan Anderson, Kenji Imadera, Sara Moradi, Tariq Rafiq

**Affiliations:** 1 Department of Space, Earth and Environment, Chalmers University of Technology, 412 96 Göteborg, Sweden; 2 Graduate School of Energy Science, Kyoto University, Uji 611-0011, Japan; imadera.kenji.7z@kyoto-u.ac.jp; 3 Laboratory for Plasma Physics, LPP-ERM/KMS, Royal Military Academy, 1000 Brussels, Belgium; saramoradi@gmail.com; 4 Department of Mechanical Engineering and Mechanics, Lehigh University, Bethlehem, PA 18015-3085, USA; rafiq@lehigh.edu

**Keywords:** plasma turbulence, gyrokinetic simulations, information length, Tsallis entropy

## Abstract

A methodology to study statistical properties of anomalous transport in fusion plasma is investigated. Three time traces generated by the full-f gyrokinetic code GKNET are analyzed for this purpose. The time traces consist of heat flux as a function of the radial position, which is studied in a novel manner using statistical methods. The simulation data exhibit transport processes with both medium and long correlation length along the radius. A typical example of a phenomenon with long correlation length is avalanches. In order to investigate the evolution of the turbulent state, two basic configurations are studied, one flux-driven and one gradient-driven with decaying turbulence. The information length concept in tandem with Boltzmann–Gibbs and Tsallis entropy is used in the investigation. It is found that the dynamical states in both flux-driven and gradient-driven cases are surprisingly similar, but the Tsallis entropy reveals differences between them. This indicates that the types of probability distribution function are nevertheless quite different since the higher moments are significantly different.

## 1. Introduction

Turbulence in magnetically confined plasma is still a popular research field due to the high impact of heat transport in fusion-related plasma [1]. This corroborates the need to investigate large-scale transport events such as bursts, streamers, blobs and avalanches [2,3,4,5,6,7,8,9,10,11]. These heterogeneous structures occur intermittently and may have a significant impact on the transport to the edge. Furthermore, simulation efforts have been suggested with the aim to investigate the effect of blobs and avalanching. The simulations indicate that blobs may alleviate the local heat flux because they enlarge the plasma-wetted area of the limiter/divertor targets. On the other hand, the prospect of avalanching is concerning and warrants further investigation. Avalanches are characterized by rapid spatial diffusion and convection of turbulence to the edge.

To investigate a broader perspective of transport induced by meso- and large-scale structures, including avalanching, global flux-driven models are needed where the profile is self-consistently determined and the radial gradients develop over time in contrast to fixed gradient-driven systems. In the global simulation framework, intermittent events, which exhibit temporal structures with frequent bursts and radial coherence, may be supported. Such transport events are called avalanches [11]. The likelihood of intermittent transport events may be studied through the properties of the probability distribution function (PDF). Intermittent transport is often characterized by unimodal PDFs with elevated exponential tails compared to a Gaussian distribution. An comprehensive analytical theory has been presented that elucidates the properties of intermittent transport of heat flux in magnetically confined plasma [12]. A significant breakthrough would be, for instance, to be able to mitigate or control of edge heat flux loads, which depend on the instantaneous amplitude of fluctuations. The elevated tails of the heat flux PDFs is a manifestation of meso- or large-scale coherent structures mediating transport [4,13]. The statistical intermittency is quantified by higher-order cumulants, e.g., the skewness and kurtosis, of the PDFs [12].

In plasma transport exhibiting meso- or large-scale events, long-range correlations may be expected. For independent processes or systems determined by mean field theory, Boltzmann–Gibb statistical mechanics is sufficient; however, for systems with long-range correlations, a more general approach is needed. Tsallis statistics are now widely applied, e.g., to solar and space plasma such as the heliosphere magnetic field; see Ref. [14]. Intermediate states between the Gaussian and Lévy distributions can be found in non-extensive statistical mechanics, which provides distributions determined by a continuous real parameter *q*; see Ref. [14]. In previous work, it was indicated that such processes should be detectable using the Tsallis entropic function, which accentuates the tail parts of the distributions.

One other possible option for detecting large-scale events is to work with the different metrics for the thermodynamic length [15,16,17,18,19,20] and the information length [21,22], which is the generalization to non-equilibrium systems. A possible solution is to use the PDFs to construct the Fisher metric in statistical space, enabling determination of the statistical length. It is thus a geometric methodology to understand stochastic processes involved in order–disorder transition, which may be expected when long-range correlations are present. As the system evolves, the PDFs change with time, and the information length measures the total number of different statistical states that a system passes through in time [21,22]. The availability of time-dependent probability density functions (PDFs) as the system evolves enables investigations into the entropy and information length of the system over time. In comparison, entropy concerns the uncertainty or disorder for one PDF (at any time), while relative entropy compares two PDFs (e.g., at two different times). However, the information length at any time is non-zero since it captures the evolution of a system between the initial and final states. In comparison, when the initial and final states are identical, the relative entropy between them is zero. The information length is proportional to the time integral of the square root of the infinitesimal relative entropy. Note also that the concept of information length is generally applicable across different fields, allowing us to comprehensively assess different processes by the same mathematical method.

The path dependence of the information length was shown to be useful for understanding the dynamical system, in particular, the attractor structure. Moreover, the information length, relative entropy and Jensen divergence were compared, and it was shown that only the information length captures a linear geometry of a linear Ornstein–Uhlenbeck process by a linear relation between L(t→∞) and the mean position of an initial Gaussian PDF.

Therefore in this work, we analyze quasi-stationary time series of heat flux generated by the global gyrokinetic software GKNET. The simulation set-up of the heat flux as a function of time and radius is discussed in Section 2. A previous study using some of the data was published in Ref. [23], which focused on the properties of the PDF tails. In this work, the analysis is extended to the Boltzmann–Gibbs entropy, Tsallis entropy and the information length. This is explored in Section 3, which provides the indicators of coherent structures and events. Here, the information length quantifies the differences between different statistical states of the system during its evolution, whereas the entropy is used to track instantaneous changes between states. At the end of the paper, Section 4, the discussion and conclusions are presented.

## 2. Numerical Model and Set-Up

The software and one data set have been presented in an earlier paper; see Ref. [23]. In this previous work, a statistical analysis of a global gyrokinetic simulation of ion temperature gradient (ITG) mode turbulence with adiabatic electrons by the software Gyro-Kinetic-Based Numerical Experimental Tokamak (GKNET) was performed. The early development of the GKNET software was presented in [24]. In the following, a brief summary of the data is presented. The simulation framework uses a circular concentric tokamak configuration with $R_{0}/a=2.79$, $a/\rho_{ti}=150$ and $q\left(r\right)=0.85+2.18{\left(\frac{r}{a}\right)}^{n}$. Here, n=2 in the normal case. The initial plasma parameters at r/a=0.5 are ${\left(R_{0}/L_{n}\right)}_{r=a_{0}/2}=2.22$, ${\left(R_{0}/L_{Ti}\right)}_{r=a_{0}/2}=10.0$, ${\left(R_{0}/L_{Te}\right)}_{r=a_{0}/2}=6.92$ and $\nu_{★}=0.28$, respectively. In this configuration, simulation parameters are taken as follows: the time step length is $\Delta t=2\times 10^{-3}R_{0}/v_{ti}$, and the grid number and system size are $\left(N_{r},N_{\theta },N_{\phi },N_{v_{\left|\right|}},N_{\mu }\right)=(128,128,64,$64,16) and $\left(L_{r},L_{\theta },L_{\phi },L_{v_{\left|\right|}},L_{\mu }\right)=\left(150\rho_{i},2\pi ,\pi ,12v_{ti},18v_{ti}^{2}/B_{0}\right)$, respectively. Note that a 1/2 wedge torus is employed in this simulation. Figure 1 in Ref. [23] shows (a) initial density, temperature and safety factor profiles and (b) deposition profiles of $A_{src}$ and $A_{snk}$. Here, the source and sink parameters are chosen to avoid large deviations from the Maxwellian distribution in the heating region and possible non-physical oscillations triggered by the fixed outer boundary condition. Within this framework, we performed gyrokinetic simulations of flux-driven toroidal ITG turbulence with external heat input $P_{in}=16$ [MW]. Note that not only turbulence and zonal flow but also the neoclassical transport and mean flow determined self-consistently by evolving equilibrium profiles can be properly traced in this framework. An investigation of the statistical properties of the heat flux in these simulations was performed in Ref. [23], which was centered around the base case with parameters similar to those of the cyclone base case, i.e., $a/\rho_{i}=150$, $a/R_{0}=0.36$ and $\tau_{snk}^{-1}R_{0}/v_{ti}=0.25$.

**Figure 1:**
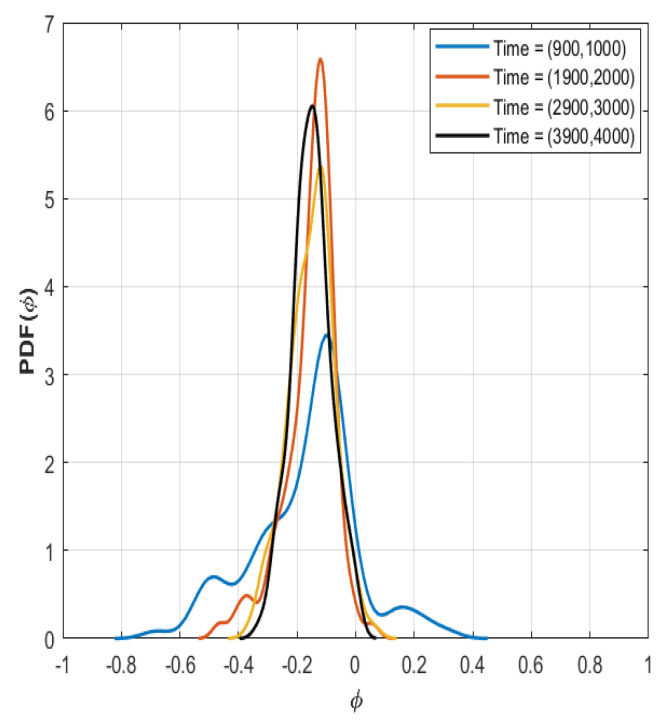
The time evolution of PDF of the electrostatic potential (ϕ) at mid radius displayed at four different time windows.

In Ref. [23], the tails of the PDFs were analyzed, and the time traces of heat flux were processed to retain their stochastic parts with only the help of Box–Jenkins modeling to remove deterministic autocorrelations. The time evolution of the PDF is shown at four instances in Figure 1; here, the change with time is apparent, and evidently this impacts the micro-turbulent properties, although it is difficult to discern and immediately connect PDFs to a change in the dynamics such as one that may be expected of a meso- or large-scale event unless careful investigation of the PDF tail properties is performed. It is concluded that this serves as a useful test case for both the previously developed methodology and for exploring entropic methods.

The accuracy of the modeled PDFs can be evaluated by comparing higher statistical moments (kurtosis) of the PDFs where a good representation was found. Previous local gyrokinetic simulations, as shown in Ref. [12], found similar probability density functions (PDFs). However, due to the global nature of the model, these simulations introduced consistently different non-Gaussian features, such as stretched exponential and Laplacian PDFs. Thus, significant heat is mediated by coherent structures such as blobs/coherent structures or streamers/avalanches. In the simulations, large, avalanche-like structures are present as an indication of this mode of transport. Although we analyzed time traces three times longer than the original simulations (which had 4000 time steps), we obtained similar results.

**Figure 2:**
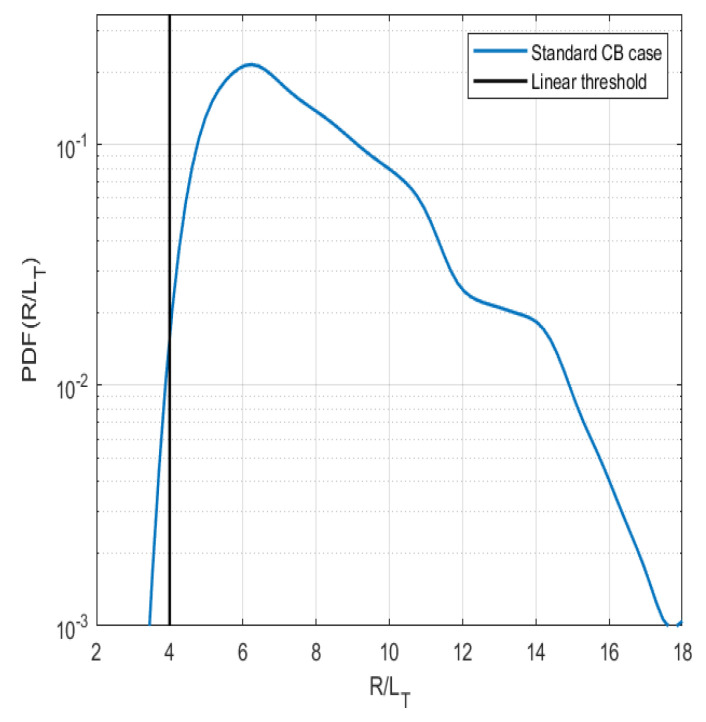
The PDF of the temperature gradient ($R/L_{Ti}$) at mid radius $a/\rho_{i}=75$.

In order to understand the properties as a function of time of the numerically generated time traces, a study of a few key indicators was performed in addition to the work of Ref. [23] where the PDFs of heat flux were investigated. In the flux-driven simulations, the radial profile self-consistently evolves over time, and the PDFs are determined by a time window of 100 samples in the time trace. It is indicated that these are characterized by fluctuations above marginal stability, and the linear stability limit is adopted from a fluid model [25] in the temperature gradient ($R/L_{Ti}$) of that profile. The likelihood of instability is shown in Figure 2, where the PDF of the temperature gradient scale length is displayed. More importantly, it is evident that the plasma is in the unstable region for most of the time during the simulation in the standard case.

In the following, X will denote the sample time trace with elements $X_{1},X_{2},\ldots ,X_{n}$. Another quantity of interest is the Hurst exponent [26] of X. In general, 0<H<1 but if H=0.5, the series is considered random (uncorrelated); if H>0.5, the series has a long-term positive autocorrelation, meaning that high (low) values in the series X will have a higher probability of being followed by another high (low) value. Conversely, if H<0.5, in the long run, with high probability, high (low) values in X will have a higher probability of being followed by another low (high) value. The Hurst exponent is calculated by the rescaled range (RS) method as the exponent *H* such that $E\left[R\left(n\right)/S\left(n\right)\right]=Cn^{H}$ for n→∞, where *C* is a constant, $E\left[x\right]$ is the expected mean, $S\left(n\right)$ is the standard deviation of the series $X_{1},X_{2},\ldots ,X_{n}$, and $R\left(n\right)$ is the range of the *n* cumulative deviations from the mean; that is, $R\left(n\right)=\max\left(Z_{1},Z_{s},\ldots ,Z_{n}\right)-\min\left(Z_{1},Z_{s},\ldots ,Z_{n}\right)$, $Z_{j}=\sum_{i=1}^{j}\left(X_{i}-m\right)$, $m=\left(\sum_{i=0}^{n}X_{i}\right)/n$. Then, *H* is calculated as the slope of the line that fits the $\log\left(R(n)/S(n)\right)$ data as a function of $\log\left(n\right)$.

**Figure 3:**
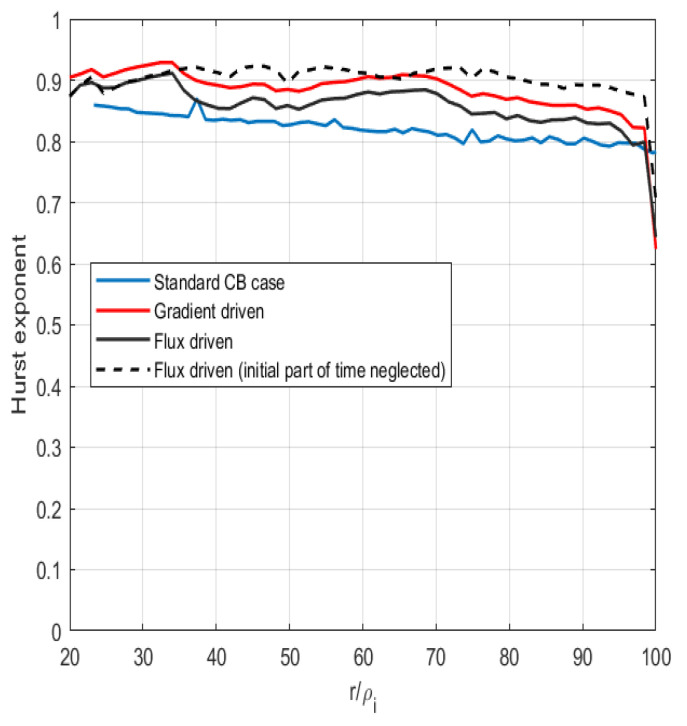
The Hurst exponent of heat flux as a function of the radial coordinate displayed for the three cases, standard, gradient-driven and flux-driven. The gradient-driven and flux-driven (at 4 MW) cases are obtained from a later iteration of GKNET.

In Figure 3, high values of the Hurst exponent are displayed. It is evident that the physical process favors repeated values. This was shown to be of importance in Ref. [27], which indicates that cumulative changes in the dynamical time may be visible as rapid increases in the information length and thus indicate a change in the state of turbulence.

## 3. Results

In this section, the entropy and information length will be computed. Both methods rely on the accuracy of estimating the turbulent state defined by the PDF. Moreover, it is expected that important information is stored in the tail part of the PDF; this thus indicates that sampling of the data is crucial. In this work, the sampling of the time series is determined by the window length $W_{L}$. The window length needs to be adjusted to match the physical processes of interest. Specifically, there should be enough data points to capture the skewness and kurtosis (i.e., the third and fourth moments of the distribution) while remaining short enough to capture the dynamics of the relevant processes. If the window length is too long, processes occurring on a shorter time scale will be obscured and not fully captured. The PDFs are thus computed at the midpoint of $W_{L}\in 2k+1$ (where *k* is an integer) and at each time instance after k+1 at the start of the time trace until k+1 samples from the end.

The dynamic time $\tau_{i}\left(t\right)$ of the $X_{i}$ subsequence estimates the instantaneous change and is computed as

(1) $$\tau_{i}{\left(t\right)}^{2}=1/\int dX_{i}\frac{1}{p\left(X_{i},t\right)}{\left(\frac{\partial p\left(X_{i},t\right)}{\partial t}\right)}^{2}.$$

More information is found in Refs. [27,28]. The information length, $L\left(t\right)$, can be directly obtained from the dynamic time by time integration:

(2) $$L\left(t\right)=\int_{0}^{t}ds\frac{1}{\tau_{i}\left(s\right)}.$$

The implementation of Equations (1) and (2) are discrete and estimated by summations and discrete differentiations, respectively, since $X_{i}$ and *t* are discrete. In this work, special attention is given to entropy and information length computed by the time dependent PDFs. It should be noted that one of the crucial quantities that characterizes out-of-equilibrium systems (e.g., turbulence) is temporal change in PDFs. The information length is sensitive to this temporal change since it is based on how quickly PDFs change in time (see Equation (2)). Physically, τ in Equation (1) gives us a characteristic time scale of a PDF. The time integral in Equation (2) then picks up the intermediate dynamics between the initial and final states (at time 0 and time t) and is obtained by measuring the clock time (dt) in unit of the instantaneous time scale τ(t). Specifically, it tells us the total number of statistically different states that a system passes through in time along the path. A unique specialty of the information length is this path dependence. Each contribution stemming from integration of the dynamic time is positive; thus, the information increases as the system evolves and changes state. It is also dependent on the path in the phase space, which indicates that the history determines the information length and would give a good estimate of changes in the state.

**Figure 4:**
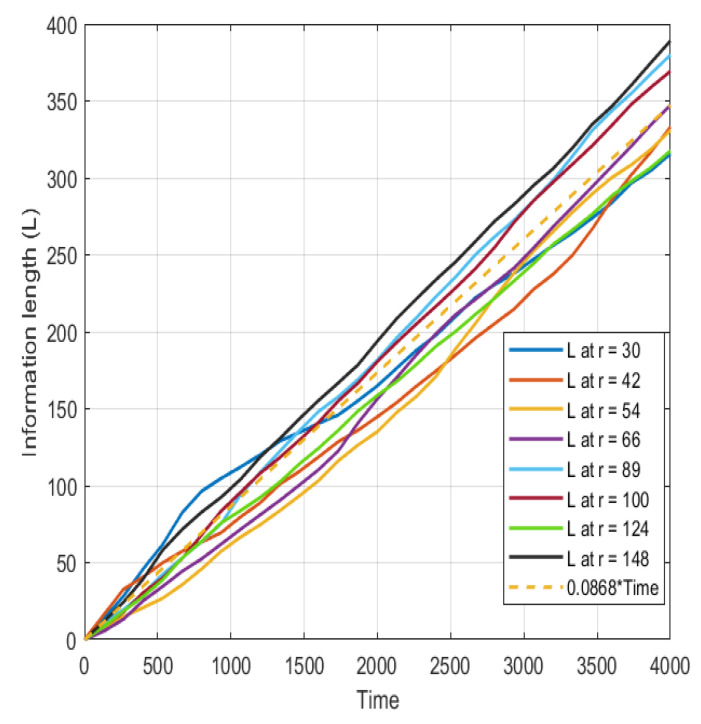
The information length *L* at different radial positions with a linear fit (dashed line).

In Figure 4, the information length computed by Equation (2) at different radii relevant for the source, core and edge regions is displayed. It is found that the information increases almost linearly over the time trace; interestingly, the information in the outboard side of the simulation domain is increased in comparison to the inboard side. This indicates the effect of generation of structures on the information length. The structures appear and disappear intermittently, reflecting an increase in the information length due to the differences in the PDFs of the different states.

In Figure 4, the information length is almost linearly growing. A linear fit (dashed line) is included for comparison. The information length is here computed with running PDFs with a window length of 200 time steps.

The information length appears to grow almost linearly, with interesting variations arising from the generation of coherent structures. This suggests that further investigation into the integrated parameter, dynamic time, would be worthwhile. The recent literature has derived analytical probability density functions (PDFs) for many different dynamical systems, including a system with a logarithmic non-linear quantity. Although the objective of Ref. [29] was different, the results are generally applicable.

Logarithmic non-linearities are found in various models, including the logarithmic non-linear Schrödinger equation (LNLSE), which is mathematically appealing because it supports solitary wave solutions (Gaussons) while retaining many simple features of linear equations. Ref. [29] implies that there is a statistical structure in the generation of dynamic time and entropy. In that work, a dynamical equation of the form was considered:

(3) $$\begin{array}{c} \frac{d\zeta (x,t)}{dt}+c\zeta \left(x,t\right)\log\left(|\zeta \left(x,t\right)|\right)+\eta \nabla^{2}\zeta \left(x,t\right)=f, \end{array}$$

where is ζ(x,t) is a smooth function of the dynamical system, *c* is a constant, η is a damping term and *f* is the forcing. For simplicity, the statistics of the forcing is assumed to be Gaussian with a short correlation time modeled by the delta function. The model Equation (3) representing the system leads to a likelihood distribution for ζ or is in the form of a probability distribution function with ζ as the variable, p(ζ):

(4) $$\begin{array}{ccc} p(\zeta ) & ∼ & e^{-\frac{\zeta }{\alpha }\left(\ln\frac{\zeta }{\alpha }-1\right)}. \end{array}$$

**Figure 5:**
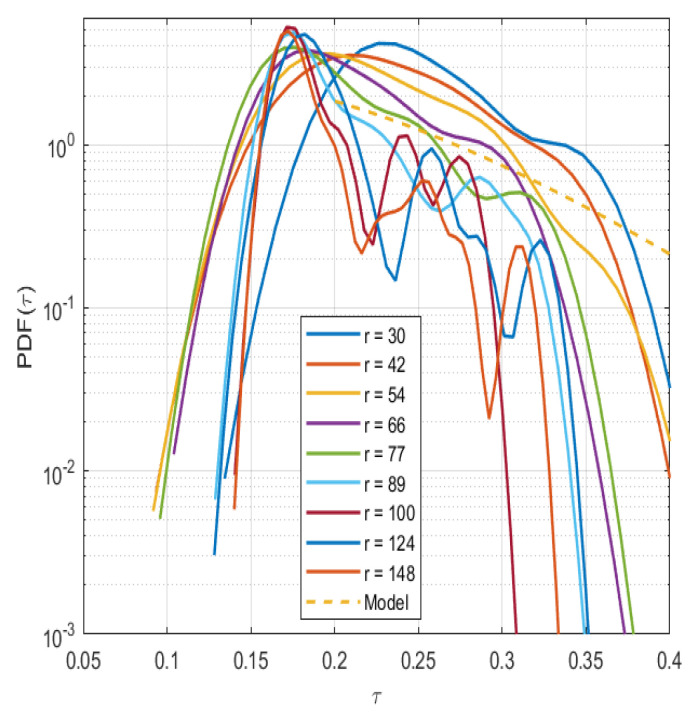
The PDF of the dynamic time τ at different radial position with a model fit (dashed line) using Equation (4).

Note that the exponential scaling of the PDF is uniquely determined by the non-linear term in the dynamical Equation (3). Although the data are scarce, it is found that the numerical scaling of the generated dynamical time in the simulation roughly follows the theoretical scaling of entropic systems. The scarcity of the data creates the non-smoothness in the PDFs; however, this is also indicated in the similarity of all scalings of the information length that mostly grow in an almost linear manner. Note that the dynamic time acts as the time unit in the statistical space, but more importantly, it measures the correlation time over which the probability density function (PDF) changes (Figure 5).

Next, the remaining part of the paper is dedicated to the data sampled from the new version of GKNET; see Ref. [30]. The main difference in the data is that, in the new data, a distinct comparison between a gradient-driven and a flux-driven case is made. For both data sets, a sixth-order safety factor $q\left(r\right)=0.85+2.18{\left(\frac{r}{a}\right)}^{6}$ is utilized in combination with a smaller radial cross-section of $a_{0}/rho_{t}i=100$. In the gradient-driven case, the initial profile evolves over time, yielding decaying turbulence, whereas in the flux-driven case additional power is used as input $P_{in}$ = 4 MW. Note that the updated software enables studies with kinetic electrons; however, here, only adiabatic electrons are retained.

**Figure 6:**
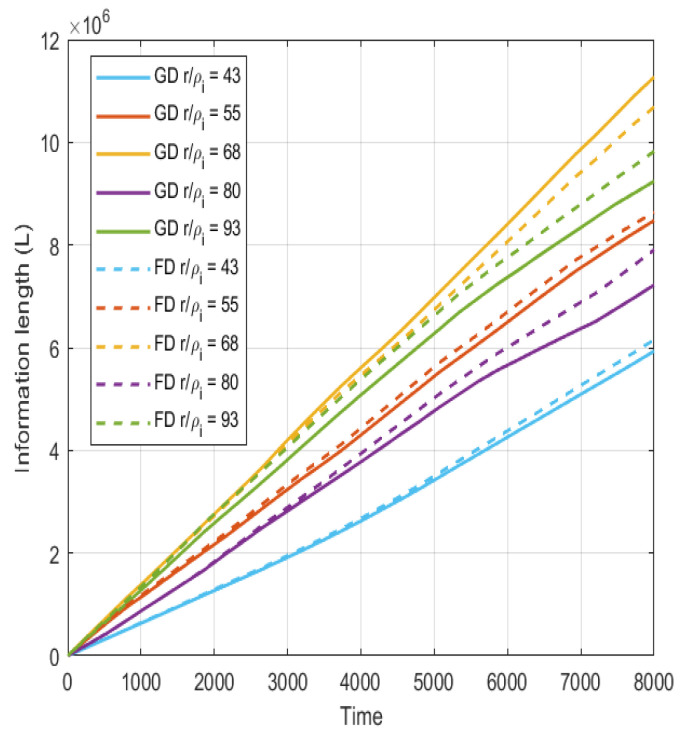
The information length *L* at different radial positions for the gradient-driven (solid lines) and flux-driven (dashed lines) cases, respectively.

Although a significant difference in the turbulence should be expected, the time evolution of the simulation is remarkably similar since the information lengths, as seen in Figure 6, closely follow each other at the different radii. It could then be concluded that similar large-scale structures are generated regardless of the differences, generating a similar dynamical process; however, it is also evident that, as time passes, the differences in the information length increase. Note that it is only at the radial location $r/\rho_{i}=68$ where the gradient-driven case has higher information length over time.

In general, a measure of the number of ways a system can be arranged is denoted entropy; here, the generalized Tsallis entropy was investigated. Note that this generalized statistical mechanics *q*-entropy or Tsallis entropy has a free parameter *q* which denotes the degree of fractality. Let *p* be the probability density function. Then, the *q*-entropy can be introduced as; see [14]:

(5) $$\begin{array}{c} S_{q}\left(p\right)=\frac{1-\int dv{\left(p\left(v\right)\right)}^{q}}{q-1}. \end{array}$$

Here, it should be mentioned that the *q*-entropy is reduced (by L’Hospital’s rule) to the conventional Boltzmann–Gibbs entropy S=−∫dXlog(p(X))p(X) for Gaussian statistics, where q→1. In analyzing complex systems out of equilibrium, non-extensive statistical mechanics has a solid theoretical basis where the parameter *q* describes the degree of non-extensivity in the system.

**Figure 7:**
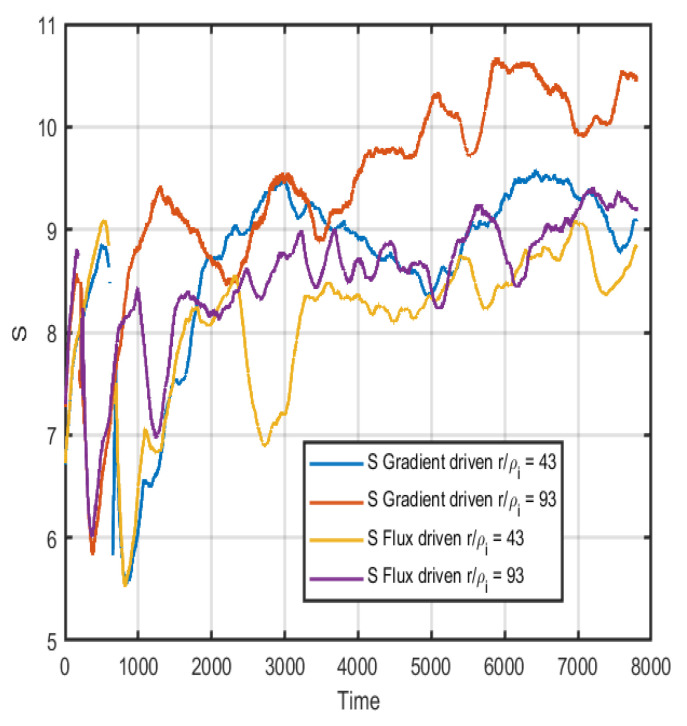
The Boltzmann–Gibbs entropy computed for the gradient and flux-driven cases at two different radii $r/\rho_{i}=43$ and $r/\rho_{i}=93$, respectively.

**Figure 8:**
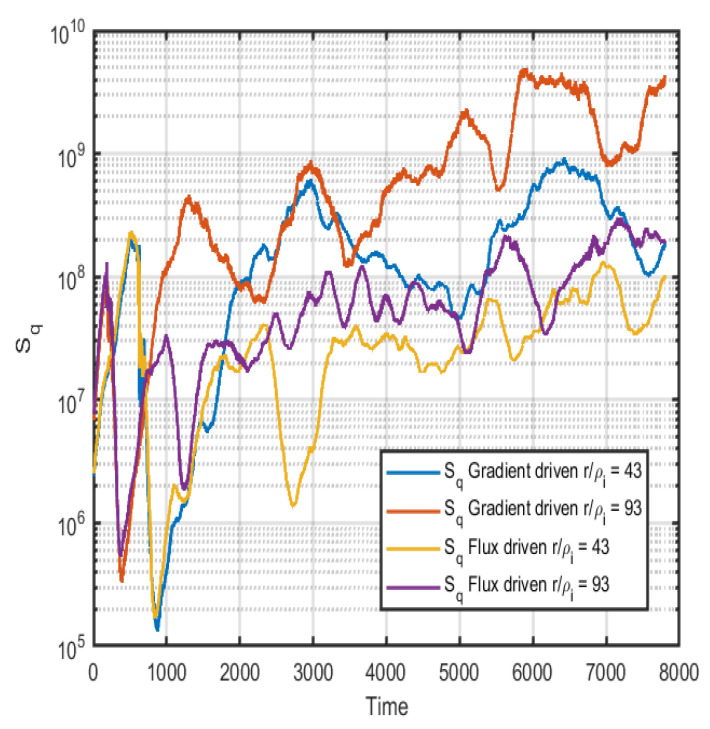
The Tsallis entropy computed for the gradient and flux-driven cases at two different radii $r/\rho_{i}=43$ and $r/\rho_{i}=93$, respectively.

Due to the similarities in the information length found in Figure 6, a direct computation of the entropy at the different time steps is performed. This should interpreted as the instantaneous entropy generated at each time step and not the total entropy in the system. The entropy is only computed using the PDFs generated with a limited time window of 200 time steps. In Figure 7, the Boltzmann–Gibbs entropy (*S*) for the gradient-driven system (blue and orange lines) and the flux-driven system (yellow and purple) at two different radii are shown. Here, the time evolution of the systems is quite similar, at least until 1000 time steps thereafter, at which point more differences can be noted; however, the order is similar, and large fluctuations are present. The Tsallis entropy is computed according to Equation (5), and the result is shown in Figure 8. In Figure 8, the Tsallis or q-entropy ($S_{q}$, with q=3.1) for the gradient-driven system (blue and orange lines) and the flux-driven system (yellow and purple) at two different radii are shown. The *q* is taken to accentuate differences in non-Gaussian properties of the PDFs; here, it is evident that at the outer radii it is around an order of magnitude difference in *q*-entropy. It is expected that the properties of the meso- and large-scale structures rest in the tail parts of the PDFs; thus, skewness and kurtosis are of interest. This gives another useful indicator of anomalous transport properties.

## 4. Summary and Conclusions

The full-f gyrokinetic code GKNET is utilized to generate heat flux time traces in several different cases. The main difference is that one case is flux-driven, and one case is gradient-driven, where the profiles are allowed to evolve according to the fluxes. This means that the gradient-driven system is more or less a decaying state where no additional energy input is used. The information length concept in tandem with Boltzmann–Gibbs and Tsallis entropy are used in the investigation of the dynamical system. In the information length concept, the probability distribution functions (PDFs) in time are computed and analyzed. The information length is obtained by computing an integral (see Equation (2)) summing up positive definite contributions over time. The information length measures the difference between two states in terms their PDFs. In comparison, for a Gaussian PDF, a statistically different state is obtained due to the mean value change (the peak position of a PDF) or due to the standard deviation (the width of a PDF). Physically, the former (mean value change) is due to the work, while the latter (standard deviation change) is due to the entropy change. More technically, for the information length, the mean value change is to be normalized by the smallest scale (standard deviation) as the width of a Gaussian PDF gives the uncertainty (error) in measuring the mean position and thus the smallest scale. In addition to the analysis by the Hurst exponent and information length, the Boltzmann–Gibbs and the Tsallis entropy are computed, and all these measures indicate different aspects of the properties of the PDFs.

It is found that although the dynamical state in the studied flux-driven and gradient-driven cases is surprisingly similar, the Tsallis entropy reveals inherent differences. This indicates that the types of probability distribution function are nevertheless quite different. In Ref. [27], time traces of gyrokinetic simulations performed by GENE were analyzed, where a combination of different metrics (information length, dynamic time and Hurst exponent) were needed to find interesting differences in physics; however, in this case, all these metrics are rather similar, making a similar analysis difficult. It seems that using direct computation of the Tsallis entropy, some aggregated information on the differences in the PDFs could be found. Moreover, a test of the PDF of dynamic time is presented that seems to roughly follow a certain scaling based on analytical estimates found in Ref. [29].

## Data Availability

The data presented in this study are available on reasonble request from the corresponding author.
